# Selective Targeting of Tumor and Stromal Cells By a Nanocarrier System Displaying Lipidated Cathepsin B Inhibitor**

**DOI:** 10.1002/anie.201402305

**Published:** 2014-06-27

**Authors:** G Mikhaylov, D Klimpel, N Schaschke, U Mikac, M Vizovisek, M Fonovic, V Turk, Boris Turk, Olga Vasiljeva

**Affiliations:** Department of Biochemistry and Molecular and Structural Biology, Jozef Stefan Institute1000 Ljubljana (Slovenia); National Research Tomsk Polytechnic University634050 Tomsk (Russia); Center of Excellence CIPKEBIP1000 Ljubljana (Slovenia); Faculty of Chemistry and Chemical Technology, University of Ljubljana (Slovenia),Center of Excellence NIN1000 Ljubljana (Slovenia); Fakultät für Chemie, Universität Bielefeld33615 Bielefeld (Germany); Department of Condensed Matter Physics, Jozef Stefan Institute1000 Ljubljana (Slovenia); Jozef Stefan International Postgraduate School1000 Ljubljana (Slovenia)

**Keywords:** cancer, cathepsin B, drug delivery, theranostics, tumor microenvironment

## Abstract

Cathepsin B (CtsB) is a lysosomal cysteine proteinase that is specifically translocated to the extracellular milieu during cancer progression. The development of a lipidated CtsB inhibitor incorporated into the envelope of a liposomal nanocarrier (LNC-NS-629) is described. Ex vivo and in vivo studies confirmed selective targeting and internalization of LNC-NS-629 by tumor and stromal cells, thus validating CtsB targeting as a highly promising approach to cancer diagnosis and treatment.

Tumor progression is orchestrated by crosstalk between different cell types in the tumor and the surrounding tissue. Tumor cells themselves can induce their adjacent stroma cells to form a permissive and supportive microenvironment for tumor progression.[1] Therefore, simultaneous targeting of the two major components of the tumor microenvironment, tumor and stromal cells, is emerging as one of the most potent strategies for the targeted therapy of cancer and may greatly increase the effectiveness of traditional anticancer therapies.[2] Furthermore, tumor progression is strongly associated with dysregulated and increased proteolysis,[1b, 3] which represents another hallmark of cancer. Most cancer-associated proteases are extracellular enzymes and some of them are also known to be secreted by normal cells.[3b, 4] Lysosomal cysteine proteases (i.e. cysteine cathepsins), however, are localized in the endolysosomal vesicles of healthy cells, and they translocate to the cell surface and/or are secreted into the extracellular tumor milieu during cancer progression. Moreover, cysteine cathepsins are expressed by both cancer and stroma cells,[5] thus making them potent cancer-specific targets.

Among cysteine cathepsins, cathepsin B (CtsB) is the most abundantly expressed and was found to be secreted by various types of cells in the tumor microenvironment, including tumor cells, tumor-associated macrophages, fibroblasts, osteoclasts, T lymphocytes, neutrophils, and endothelial cells.[6] Furthermore, secreted CtsB was shown to be associated with the plasma membrane through binding to the cell surface through the light chain of the annexin II tetramer, p11, and colocalizing in caveolae.[7] Overexpression and secretion of CtsB has been demonstrated in different types of cancer, including breast cancer, melanoma, glioma, and cancer of the esophagus, pancreas, colon, and prostate, thus supporting its use as a universal target for drug delivery across a broad spectrum of indications.[5b–d]

In this study, we aimed to develop a drug delivery system that targets extracellular CtsB through a specific CtsB inhibitor conjugated to a highly biocompatible liposomal nanocarrier. The construct was thus designed for the selective targeting of CtsB-overexpressing tumor and stromal cells in the tumor microenvironment. We initially synthesized a lipidated form of the CtsB inhibitor NS-629 through the conjugation of 1,2-distearoyl *sn*-glycero-3-phosphoethanolamine-*N*-[carboxy (polyethylene glycol)-2000] (DSPE-PEG(2000) carboxylic acid) to H_2_N-(CH_2_)_6_-HN-Gly-Gly-Leu-(2*S*,3*S*)-*t*Eps-Leu-Pro-OH[8] (Scheme 1, Figure 1 a). The protected CtsB inhibitor **2** was coupled to DSPE-PEG(2000) carboxylic acid by using EDC and HOBt to yield the protected lipidated inhibitor **3**. The protecting group was removed by treating **3** with 95 % aqueous TFA. NS-629 (**1**) was obtained in two steps with a yield of 63 % and was fully characterized by TLC and HRMS. The non-lipidated inhibitor NS-134, the precursor of NS-629, binds to the active form of CtsB by forming a covalent bond with the catalytic Cys29 residue and occupies the S4 to S2’ substrate-binding sites.[9] The efficacy of the lipidated NS-629 inhibitor was evaluated by active-site titration with recombinant CtsB. The inhibitor bound to CtsB with an apparent 1:1.4 stoichiometry, thereby resulting in irreversible and selective inactivation of CtsB (Figure 1 b and Figure S1 in the Supporting Information). These results demonstrate the potential of the NS-629 inhibitor to be used as a ligand for the active targeting of CtsB.

**Figure 1:**
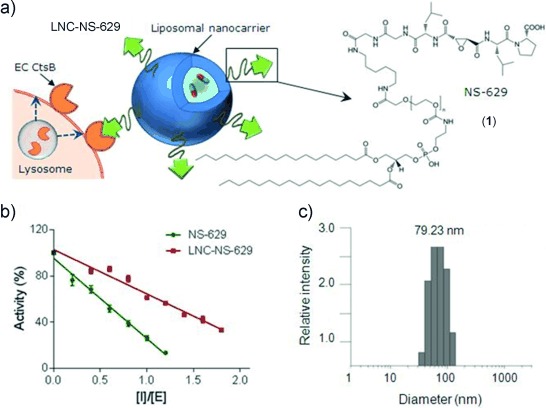
The design and characterization of LNC-NS-629. a) A Scheme showing the liposomal nanocarrier conjugated to the lipidated CtsB inhibitor (NS-629) to form the LNC-NS-629 drug delivery system, which is capable of targeting extracellular (EC) CtsB. b) Titration of CtsB with NS-629 and LNC-NS-629 at pH 6.0 and 25 °C. The solid lines were generated by linear regression analysis. c) DLS measurements showing the distribution of diameters of the LNC-NS-629 nanoparticles and their average size (*D*=79.23 nm).

**Scheme 1:**
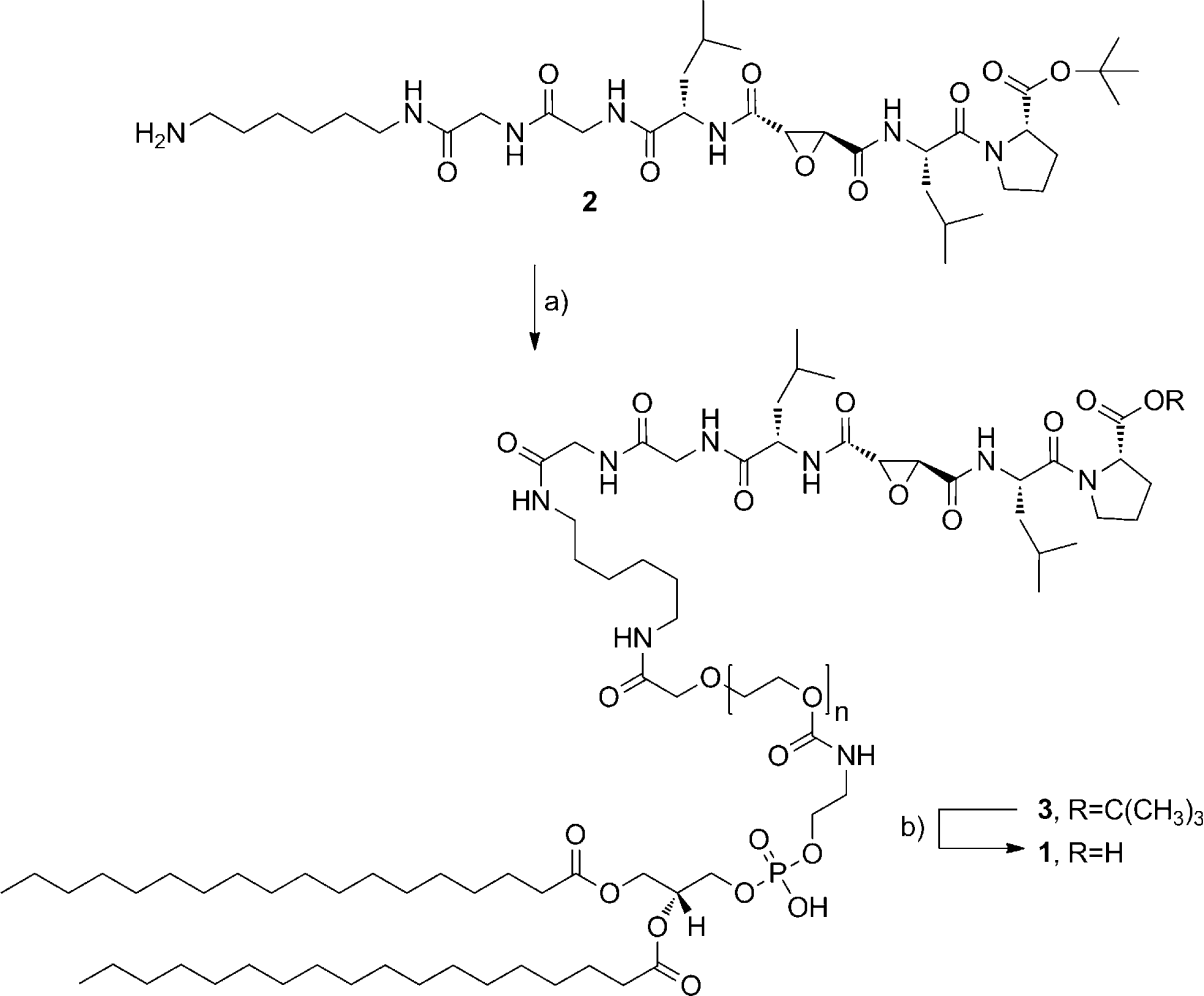
Synthesis of the lipidated CtsB inhibitor NS-629. Reaction conditions: a) DSPE-PEG(2000) carboxylic acid (*n*=41–56), EDC, HOBt, CHCl_3_, 48 h, 25 °C; b) TFA/H_2_O (95:5, *v*/*v*), 1 h, 25 °C. EDC=1-(3-dimethylaminopropyl)-3-ethylcarbodiimide hydrochloride, HOBt=1-hydroxybenzotriazole, TFA=trifluoroacetic acid.

Liposomes are nanosized phospholipid vesicles that have been widely recognized as one of the most potent drug delivery vehicles.[2a, 10] They are biologically inert and have considerable structural and pharmacokinetic advantages for drug delivery, thus leading to a reduction of the toxic effects of the encapsulated compounds and enhancement of the therapeutic efficacy of certain drugs.[2a, 10] These advantages have resulted in several formulations in regular clinical use. To enable the CtsB-targeted delivery of diagnostics or therapeutics to the cancer site, NS-629 was incorporated through a lipid linker into the preformed sterically stabilized polyethylene glycol (PEG)-coated nanosized stealth liposomes, thereby forming a lipidated nanocarrier (LNC) capable of specific CtsB targeting (LNC-NS-629; Figure 1 a). The liposome surface was PEGylated to reduce opsonization of the liposomes and their subsequent clearance by the reticuloendothelial (mononuclear phagocyte) system.[2a] The LNC-NS-629 particles appeared under atomic force microscopy (AFM) as nanospheroids (Figure S2) with an average diameter of 79.23 nm as measured by dynamic light scattering (DLS; Figure 1 c). Active-site titration with recombinant CtsB showed that LNC-NS-629 was 38 % active, which presumably reflects an even distribution of the inhibitor molecules between the interior and exterior of the liposome (Figure 1 b).

To evaluate the efficacy of the CtsB-targeting liposome delivery system, we first verified that CtsB is expressed and secreted by the primary tumor cells derived from a genetically engineered mouse model of breast cancer (MMTV-PyMT)[6d and by the primary mouse macrophages. Bone marrow derived macrophages were isolated from mice and differentiated into M2 phenotype macrophages (dBMMs) through culture in L929-cell conditioned media, which contains macrophage colony stimulating factor (M-CSF). The proteomic study performed on conditioned media from primary tumor cells and differentiated macrophages revealed a high level of extracellular CtsB secreted by both tumor cells and dBMMs (Table 1 and Table S1 in the Supporting Information). Relative quantification of secreted proteins by spectral counting showed that CtsB was the most abundant protease secreted from differentiated macrophages. Furthermore, membrane-bound CtsB was detected on PyMT tumor cells by using a cell surface labeling approach[6d] with biotinylated inhibitor NS-196 (Figure S3). Collectively, these data support the use of CtsB as a target for theranostic applications in cancer.

**Table 1:** A list of proteins identified in conditioned media from primary PyMT tumor cells (PyMT TC) and differentiated bone marrow derived macrophages (dBMM).The proteins are ordered by relative abundance as estimated from spectral counting in tumor cells.

Protein	Uniprot accession	Number of peptides	Peptide spectral counts	
			PyMT TC	dBMM
Cathepsin B	P10605	15	34	94
MCF-1	P07141	5	20	4
Cathepsin L	P06797	9	16	8
MMP-12	P34960	10	0	11
Ferritin light chain	Q3THE6	8	0	22

Next, the specificity of LNC-NS-629 binding to extracellular CtsB was validated in a cellular model by using primary MMTV-PyMT cells as tumor cells and dBMMs as stromal cells. To prevent passive endocytosis, the cells were precultured at low temperature (4 °C) followed by incubation with LNC-NS-629 labelled with Alexa Fluor 555. Lipidated nanocarriers lacking NS-629 (LNC) were used as a negative control. After extensive washing of the cells to remove unbound liposomal constituents, significantly higher fluorescence was measured in the PyMT tumor and dBMM cells treated with LNC-NS-629 than in cells incubated with naked LNC control liposomes (Figure 2 a, b) or in undifferentiated macrophages (Figure S4 a). Moreover, fluorescence microscopy revealed very effective internalization of the labeled LNC-NS-629 and its compartmentalization in endosomal vesicles by both types of cells (Figure 2 b). Furthermore, no difference between targeted (LNC-NS-629) and untargeted (LNC) internalization of the encapsulated dye was detected in normal cells, such as mouse embryonic fibroblasts (Figure S4 b, c). Taken together, these results demonstrate a strong potential for the use of LNC-NS-629 as a drug delivery system that is selectively targeted to tumor cells and tumor-associated macrophages.

**Figure 2:**
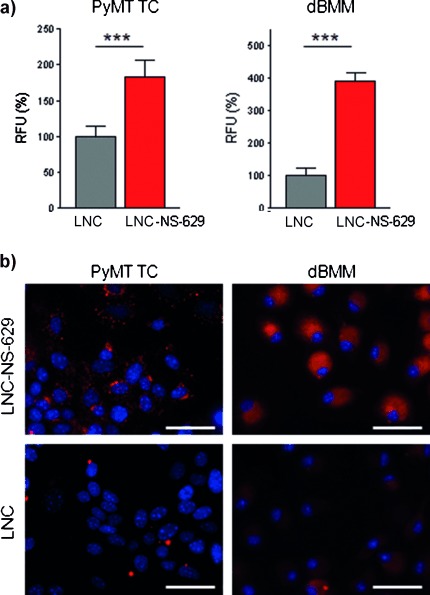
Ex vivo validation of the targeting and release of LNC-NS-629. a) Measurement of the accumulated fluorescence signal after the incubation of primary PyMT tumor cells (PyMT TC) and bone marrow derived macrophages (dBMM) with Alexa Fluor 555 functionalized naked LNC and LNC-NS-629 for 1 h at 4 °C. The data points represent the mean of three separate experiments. *** indicates p<0.001, RFU=relative fluorescence units. b) Fluorescence images of primary PyMT tumor cells (PyMT TC) and bone marrow-derived macrophages (dBMM) incubated with naked LNC or LNC-NS-629 functionalized with Alexa Fluor 555 for 1 h at 4 °C. Scale bar: 20 μm. The images are representative of three separate experiments.

To validate the efficiency of targeting to the tumor microenvironment in vivo and to investigate the potency of the CtsB-targeted system for diagnostic applications, we applied magnetic resonance imaging (MRI). LNC-NS-629 was functionalized through the encapsulation of Magnevist, a gadolinium-based magnetic resonance contrast agent most commonly used in clinical MRI, to yield a composite nanoprobe [NLP-NS-629-(Gd)] that enhances the MRI signal primarily by decreasing the spin-lattice relaxation time (*T*_1_) in tissues in which it localizes. Longitudinal *T*_1_-weighted MRI following the administration of LNC-NS-629-(Gd) to mice bearing orthotopic PyMT tumors[2a] showed significant enhancement of contrast at the tumor site and delineation of the tumor boundary only 1 h post-administration (Figure 3 a). Moreover, the contrast signal enhancement of the tumor measured 24 h after treatment resulted in a high tumor-to-background signal ratio. Notably, no accumulation of NLP-NS-629 nanoparticles was detected in normal tissues, as confirmed by MRI scanning of healthy mice administered with LNC-NS-629-(Gd) (Figure S5). Furthermore, the ability to sustain imaging for a significantly longer time compared to conventional contrast agents provides evidence for successful internalization of the cargo of NLP-NS-629, thus supporting its use as a drug delivery system. Likewise, LNC-NS-629 may also be used in developing novel strategies for cancer-targeted diagnostic imaging in clinical oncology.

**Figure 3:**
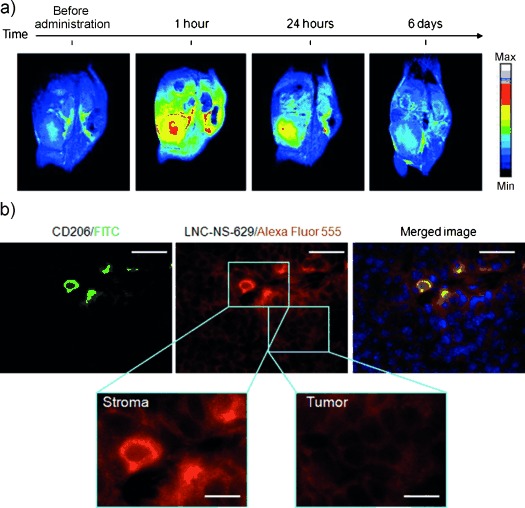
The targeting of LNC-NS-629 in a mouse breast cancer model in vivo and ex vivo. a) *T*_1_-weighted MR images (TE=12 ms, TR=400 ms) of an orthotopic transplanted breast cancer mouse before and 1, 24, and 144 h after the administration of LNC-NS-629 containing the MRI contrast agent Magnevist. The bright signal 1 and 24 h after injection in *T*_1_-weighted MR images indicates the successful targeting of Magnevist-loaded LNC-NS-629 to the tumor site. b) Uptake of LNC-NS-629 functionalized with Alexa Fluor 555 (red) by tumor cells and tumor-associated macrophages after intravenous injection of LNC-NS-629. Tissues were co-stained for a marker for tumor-associated macrophages (CD206-FITC; green). The merged image also shows the cell nuclei, which were stained with DAPI (blue). DAPI=4’,6’ diamino-2-phenylindole. Scale bars: 100 μm, enlarged pictures 10 μm.

To confirm the targeting of the LNC-NS-629 cargo to the tumor and its microenvironment at the tissue level, mice bearing orthotopically transplanted congenic mammary tumors were treated with LNC-NS-629 loaded with Alexa Fluor 555. Upon examining tumor sections by fluorescence microscopy, a significant accumulation of fluorescent LNC-NS-629 was detected in both malignant tumor cells and stromal cells of the tumor microenvironment (Figure 3 b). No targeting of tumor cells was detected when using labeled LNC, whereas the fluorescent signal detected in macrophages most probably resulted from the nonspecific phagocytosis of circulated material (Figure S6). Furthermore, LNC-NS-629 nanoparticles were successfully excreted from the body without any evident accumulation in the analyzed tissues (Figure S7), thus fulfilling another critical parameter for in vivo application.

Finally, we investigated the therapeutic applicability of the LNC-NS-629 targeted drug delivery system through an in vitro cytotoxicity study performed on PyMT tumor cells treated with the anticancer drug doxorubicin encapsulated in LNC-NS-629 or LNC nanocarriers. Notably, targeted treatment with doxorubicin encapsulated in LNC-NS-629 was 22-fold more potent in killing PyMT tumor cells than doxorubicin encapsulated in the naked LNC liposomes (IC_50_, 0.05 versus 1.1 μg mL^−1^; Figure 4).

**Figure 4:**
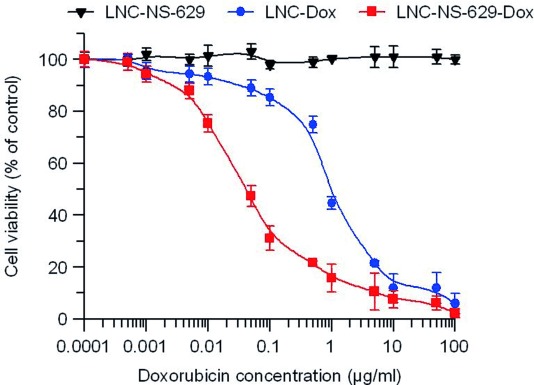
The in vitro cytotoxicity of LNC-NS-629 targeted doxorubicin in PyMT mouse breast cancer cells. PyMT cells were treated with doxorubicin encapsulated in naked LNC (LNC-Dox) and CtsB-targeted LNC-NS-629 (LNC-NS-629-Dox) liposomes at varying concentrations. Cytotoxicity was determined by using the 3-(4,5-dimethylthiazol-2-yl)-2,5-diphenyltetrazolium bromide assay. Cell viability was calculated as a percentage of viable cells.

The main challenge of any treatment is to increase the therapeutic index of the drug while minimizing side effects. The clinical utilization of most conventional chemotherapeutics is a compromise between the two factors and is limited either by an inability to deliver therapeutic drug concentration(s) to the target tissues or by severe and harmful toxic effects to healthy tissues. Despite progress in the identification of targets overexpressed in cancer cells, their expression in normal healthy tissues represents a major limitation for applications in medicine. Proteases upregulated in the tumor microenvironment thus represent a new spectrum of targets for enabling drug targeting and activation. However, whereas most of the studies focus on extracellular proteases such as the urokinase plasminogen activator system[11] or matrix metalloproteinases (MMPs),[12] the targeting of cysteine cathepsins, which are normally intracellular enzymes and are secreted by cells exclusively in pathological conditions, has not previously been investigated for theranostic applications.

Herein, we report the development of a new potent targeted delivery platform based on nanosized liposomes displaying an epoxide-based cathepsin B inhibitor conjugated to a PEG-functionalized lipid. This system leverages a unique property of the lysosomal protease CtsB, namely that it is specifically translocated to the extracellular milieu of the tumor microenvironment during cancer progression. We have demonstrated the efficiency of the LNC-NS-629 targeted drug delivery system by the successful targeting of fluorescent agents and clinical diagnostic compounds to the tumor and its microenvironment. Moreover, the use of LNC-NS-629 with an encapsulated MRI contrast agent was shown to be an effective diagnostic tool, thus enabling the precise detection of a tumor through noninvasive diagnostic imaging. The combination of a selective targeting agent with a highly biocompatible liposomal carrier allows us to simultaneously encapsulate different diagnostic contrast agents. As a result, LNC-NS-629 can be used for multimodal imaging approaches for the precise detection of tumors and metastasis. Moreover, the encapsulation of both diagnostic and therapeutic agents creates the exciting potential for simultaneous targeted drug delivery and monitoring of drug distribution by different diagnostic modalities. Furthermore, because CtsB is known both to participate in tumor progression by promoting the migration and invasion of tumor cells[6d, 13] and to induce adaptive response to chemotherapy,[6c] the LNC-NS-629 system may combine the efficient targeted delivery of drugs and diagnostic agents with a sustained inhibition of CtsB by NS-629, thereby providing an additional benefit for treatment.

In conclusion, the CtsB-targeting approach described herein is a major advance in the development of highly specific, multifunctional targeted drug delivery technologies and has the potential to increase the efficacy of cancer diagnostics and treatment, either separately or in a theranostic approach.
