# Supplementary material for: Selective Targeting of Tumor and Stromal Cells By a Nanocarrier System Displaying Lipidated Cathepsin B Inhibitor
Source: Angew Chem Int Ed Engl. 2014 Jun 27;53(38):10077–81. doi: 10.1002/anie.201402305 (PMC4499245; doi:10.1002/anie.201402305)
Supplement: Supplementary file 1 [file anie0053-10077-sd1.pdf]

Angewandte Chemie
Eine Zeitschrift der Gesellschaft Deutscher Chemiker

Supporting Information

© Wiley-VCH 2014

69451 Weinheim, Germany

Selective Targeting of Tumor and Stromal Cells By a Nanocarrier System Displaying Lipidated Cathepsin B Inhibitor**

G. Mikhaylov, D. Klimpel, N. Schaschke, U. Mikac, M. Vizovisek, M. Fonovic, V. Turk, Boris Turk, and Olga Vasiljeva**

anie_201402305_sm_miscellaneous_information.pdf

Synthesis of the lipidated CtsB inhibitor NS-629. To convert the TFA salt into the free amine, H_2N-$(CH_2)_6$-NH-Gly-Gly-Leu-(2*S*,3*S*)-*t*Eps-Leu-Pro-O*t*Bu × TFA[1] (14 mg, 17 µmol) was dissolved in $CHCl_3$ (1 ml) and washed with 0.1 M NaOH (1 ml). The organic layer was separated, dried over Na_2SO_4 and filtered. In parallel, DSPE-PEG(2000) carboxylic acid (Avanti Polar Lipids, Inc.; 11.5 mg, 4 µmol) was dissolved in $CHCl_3$ (1 ml) and EDC (5.2 mg, 27 µmol), HOBt (3.0 mg, 22 µmol) and the solution of the inhibitor building block was added. After stirring for 48 h at room temperature, the reaction was completed as monitored by TLC (spots were visualized by dipping the TLC plates into a solution of $(NH_4)_6Mo_7O_{24} \times 4\ H_2O$ (50 g/l) and $Ce(SO_4)_2$ (2 g/l) in sulfuric acid (c=0.5 mol/l) followed by heating). The reaction mixture was then diluted with $CHCl_3$ to a volume of 30 ml and washed with 5% aq. $KHSO_4$ (3x), 5% aq. $NaHCO_3$ (3x) and brine (2x). The organic layer was dried over Na_2SO_4, filtered and the solvent was evaporated under reduced pressure. The residue was dissolved in acetonitrile/water (1:3 v/v) and lyophilized. The obtained coupling product (8 mg, 2.3 µmol) was dissolved in TFA/H_2O (95:5 v/v, 1 ml) and stirred at room temperature for 1 h. The solution was added dropwise to methyl-*tert*-butyl ether/petrol ether (1:1 v/v, 40 ml). The precipitate was collected by centrifugation, washed with petrol ether (3x) and dried. The lipidated CtsB inhibitor NS-629 was obtained as colourless powder; yield: (9 mg, 63% over two steps); TLC ($CHCl_3$/MeOH/AcOH 13:5:0.18, *v*/*v*/*v*) R_f 0.62; HRMS (ESI): *m/z*=3614.2182 [M]; calcd for $C_{171}H_{327}N_8O_{68}P$: 3614.2175 (given is M which was obtained by deconvolution of the observed mass signals ($[M+3H]^{3+}$, $[M+4H]^{4+}$ and $[M+5H]^{5+}$)) of the most abundant isotopomer of the most abundant signal (n=48)).

Preparation of LNC-NS-629. Lipidated nanocarrier with incorporated NS-629 ligand (LNC-NS-629) was prepared from 96.3% L-a-phosphatidylcholine (Avanti Lipids) and 3.7% lipidated inhibitor NS-629 with a total lipid concentration of 2.7 mM. Organic solvent was evaporated in an Eppendorf Concentrator 5301 (Eppendorf), resulting in the formation of dry lipid films. Their subsequent hydration

in 0.1 mM phosphate buffer, pH 6.0, led to the formation of multilamellar vesicles with incorporated NS-629 ligand. The multilamellar vesicles were extruded by a mini-extruder containing a polycarbonate membrane with pore size 100 nm (Avanti Lipids), in order to generate nanosized unilamellar bilayer liposomes forming LNC-NS-629. The morphology and size of the LNC-NS-629 was characterized by atomic force microscopy with a Nanoscope III Multimode scanning probe microscope (Digital Instruments), operated in tapping mode, and by dynamic light scattering (DLS) using a PDDLS/BatchPlus System (Precision Detectors).

For imaging studies LNC-NS-629 was functionalized with Alexa Fluor 555™ (Invitrogen) or Magnevist® (Bayer Schering Pharma). Alexa Fluor 555™ (100 µg/ml) or Magnevist® (1 mg/ml) were suspended in 0.1 M phosphate buffer, pH 6.0, containing 1 mM EDTA and 0.1% (v/v) PEG, and encapsulated in LNC-NS-629 as described. The fluorescent LNC-NS-629 was separated from free fluorescent dye or contrast agent by gel filtration on a SephadexTM G-25 M column (GE Healthcare).

Titration of cathepsins with NS-629 and LNC-NS-629. The kinetic reaction between CtsB, CtsK, CtsL, CtsS, CtsD and NS-629 or LNC-NS-629 were analyzed by continuous measurements of the loss of enzymatic activity at different concentration of compounds in the presence of fluorogenic substrates: Z-Arg-Arg-AMC for CtsB, Z-Phe-Arg-AMC for CtsK, CtsL, CtsS and Mca-Pro-Leu-NH_2 for CstD. NS-629 or LNC-NS-629 in increasing concentrations ranging from 10 to 90 nM cathepsins and 0.5 mM of dithiothreitol (DTT), were mixed in a plate with 0.1 M phosphate buffer, pH 6.0, containing 1 mM EDTA and 0.1% (v/v) PEG. After 15 minutes incubation at 37° C the kinetics of fluorogenic substrates hydrolysis was monitored continuously for 10 min by a TECAN Saphire plate reader (Tecan, Austria).

Labeling of membrane-binding cathepsin B with biotinylated inhibitor. PyMT cells were plated on six-well plate and grown to confluency. Medium was removed, and cells were incubated for 1 hour at 4˚C with ice-cold cell medium containing 50 mM HEPES supplemented with 500 nM of NS-196, the

biotinylated form of NS-134. Cells were thoroughly washed and harvested by scraping and lysed on ice in 250 mM Tris-HCl (pH 6.8) and 0.1% Triton X-100 containing 10 mM EDTA for 30 minutes. After clearing by centrifugation at 10,000 rpm g for 10 minutes at 4°C, the supernatants were used as cell lysates. Protein content was determined with DC Protein Assay (Bio-Rad Laboratories, Inc., Hercules, CA), and lysates were normalized to equal amounts of protein. Proteins were separated by SDS-PAGE, blotted onto a polyvinylidene difluoride (PVDF) membrane, and detected with streptavidin peroxidase. As molecular mass markers, prestained protein standards were used (MBI Fermentas, St. Leon-Rot, Germany).

Cell cultures preparation. Primary PyMT cells and MEFs were prepared as described before[2]. Mouse bone marrow-derived macrophages (BMMs) were isolated from bone marrow of a 12-week old male mice FVB strain. Isolated BMMs were differentiated into alternatively activated (or M2) macrophages by incubation with 15% L929 conditioned medium in DMEM medium containing 20% heat inactivated FBS, 1%PS and 1% Glutamax, as a source of M-CSF, for one week.

Proteomic analysis of PyMT and dBMMs conditioned media. The conditioned media of primary PyMT tumor cells and differentiated BMMs were generated by 24 hours incubation of cells in serum free medium. The obtained conditioned media were then concentrated 10-fold using Centricon filter units with 3000 Da cut-off (Millipore). Samples were separated on a 12.5 % precast SDS-PAGE gel (Lonza) and visualized by silver staining. Each protein lane was cut into six slices and destained. Gel slices were subjected to reduction with 10 mM DTT in 25 mM ammonium bicarbonate, followed by alkylation with 55 mM iodoacetamide in the same buffer. Gel pieces were washed twice with 25 mM ammonium bicarbonate, dried on a Speedvac and rehydrated in 25 mM ammonium bicarbonate containing 1 µg of porcine sequence grade modified trypsin (Promega). Samples were digested overnight at 37°C. Digested peptides were extracted from the gel with 50 % acetonitrile solution

containing 5 % formic acid and concentrated to 15 µl. Samples were analyzed with an LTQ Orbitrap Velos (Thermo Scientific) mass spectrometer coupled to a Proxeon-nanoLC (Proxeon) liquid chromatography unit. Peptides were loaded on a C_{18} EASY trapping column (Proxeon) and separated on a C_{18} PicoFrit™ AQUASIL analytical column (New Objective) at a flow rate of 300 nL/min. Elution was performed with a 60 minute acetonitrile gradient from 5-40% in 0.1% solution of formic acid, at a flow rate of 300 nL/min. Nine most intense precursor ions in each full scan were selected for CID fragmentation. Dynamic exclusion was set at a repeat count of 1 with exclusion duration of 60 s. Database searches were performed against IPI mouse database using the MaxQuant software[3]. Carbamidomethylation of cysteines was set as a fixed and oxidation of methionines as a dynamic modification. Relative quantification of identified proteins was performed using spectral counting with at least 2-fold difference in spectral count being considered as significantly different.

Assessment of LNC-NS-629 internalization *ex vivo*. MCF-7, THP-1, MEFs, primary PyMT tumor cells and dBMMs were cultured at 4° C for 30 minutes to block active endocytosis, followed by incubation with 0.74 mM Alexa Fluor 555-functionalized naked LNC or LNC-NS-629 for an additional hour. Next, cells were washed with PBS and fluorescence intensity was examined with a TECAN plate reader. For imaging, cells were stained with Hoechst 33342 (Fluka) and examined with an Olympus fluorescence microscope (Olympus IX 81) with Imaging Software for Life Science Microscopy Cell[f].

***In vitro* cytotoxicity.** PyMT mouse breast cancer cells were seeded in 96-well plates (5,000 cells per well) and incubated with LNC-NS-629 or naked liposomes loaded with doxorubicin (0-100 mg/ml) for 3 h at 4ºC. Cells were washed three times with supplemented PBS to remove drugs and incubated with fresh medium for additional 12 h at 37ºC. Cell viability was assayed using the 3-(4,5-dimethylthiazol-2-yl)-2,5-diphenyltetrazolium bromide staining, and the absorbance was read at 570 nm using TECAN Saphire plate reader (Tecan, Austria).

Animal model and *in vivo* MR imaging. FVB/N-TgN(MMTVPyVT)634Mul mice were kindly provided by Thomas Reinheckel (Institut für Molekulare Medizin und Zellforschung, Albert-Ludvig Universitat Freiburg, Germany) and used in order to obtain primary tumor cells in accordance with protocols approved by the Veterinary Administration of the Republic of Slovenia (VARS) and the government Ethical Committee. Procedures for animal care and use were in accordance with the "PHS Policy on Human Care and Use of Laboratory Animals" and the "Guide for the Care and Use of Laboratory Animals" (NIH publication 86-23, 1996). In order to generate tumors for the MRI study, primary PyMT tumor cells were obtained from 14 week old MMTV-PyMT transgenic mice as described[2], culture-expanded and suspended in 200 µl serum free Dulbecco's Modified Eagle Medium (DMEM) (Invitrogen). 5 x 10^5 cells were then inoculated into the left inguinal mammary gland of the congenic recipient mouse (FVB/N mouse strain). All MR experiments were performed on a TecMag Apollo MRI spectrometer with a superconducting 2.35 T horizontal bore magnet (Oxford Instruments) using a home-made surface RF coil. For *in vivo* detection, 200 µl of LNC-NS-629 loaded with 0.5 mM Gadopentetate dimeglumine (Magnevist®, Schering AG) were administered intravenously. Two-dimensional T_1-weighted MR images were taken using a standard multislice spin-echo pulse sequence with an echo time (TE) of 12 ms and a repetition time (TR) of 400 ms before injection, 1 hour and 24 hours post-injection. The field of view was 90 mm with in-plane resolution of 351 µm and slice thickness of 3.5 mm. During imaging, mouse was anaesthetized by subcutaneous injection of ketamine/xylazine/acepromazine (50/10/1.0 mg/kg).

Analysis of LNC-NS-629 targeted delivery *in vivo* at tissue level. Alexa Fluor 555-functionalized LNC-NS-629 (100 µg/ml) was injected intravenously to the orthotopic transplanted breast cancer model mice. 24 hours after injection tumors were resected, fixed in 10% formalin overnight, dehydrated using Shandon Tissue Processor (Shandon Citadel 1000) and molded with paraffin (Microm EC 350 Paraffin

Embedding Station). Paraffin sections were cut to 5 µm thickness mounted with anti-fade media containing DAPI (Prolong@ Gold antifade reagent with DAPI, Invitrogen) and visualized as described above. Rat anti-mouse monoclonal FITC-conjugated CD206 antibodies (1:100 AbD Serotec) were used for the detection of tumor-associated macrophages. Samples were co-stained with Hoechst 33342 (5 µg/ml, Fluka), mounted in ProLong® Gold antifade reagent (Invitrogen) and examined with an Olympus fluorescence microscope (Olympus IX 81) with Imaging Software for Life Science Microscopy Cell[f].

Statistical analysis. Quantitative data are presented as means ± standard error. Differences were compared using Student's t-test. When P-values were 0.05 or less, differences were considered statistically significant.

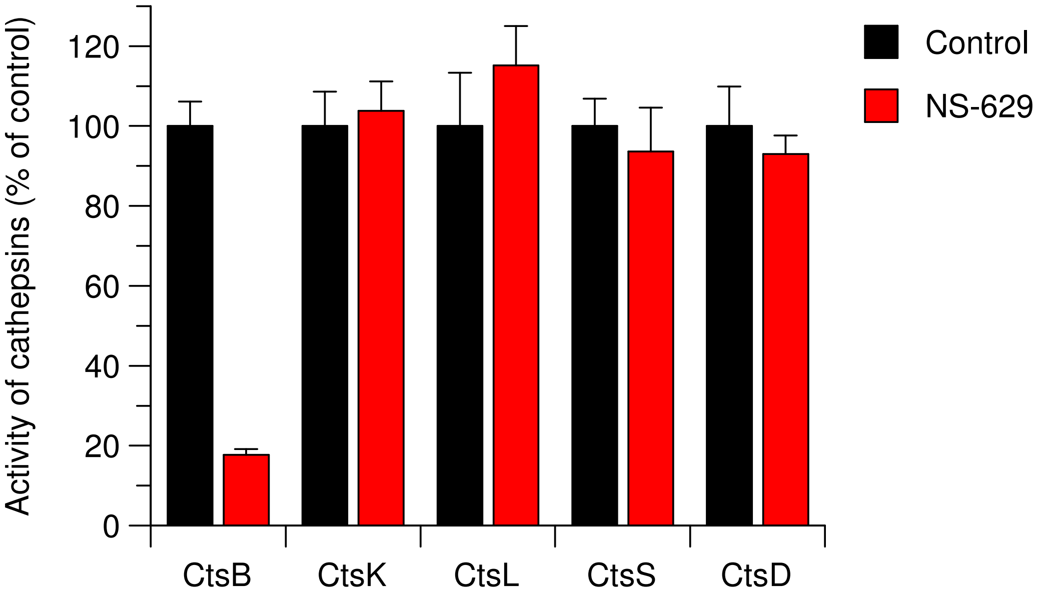

Figure S1. NS-629 selectivity for inhibition of CtsB activity. Activity of cathepsin B (CtsB), cathepsin K (CtsK), cathepsin L (CtsL), cathepsin S (CtsS) and cathepsin D (CtsD) was measured in the presence of NS-629 inhibitor at pH 6.0 and 25 ℃.

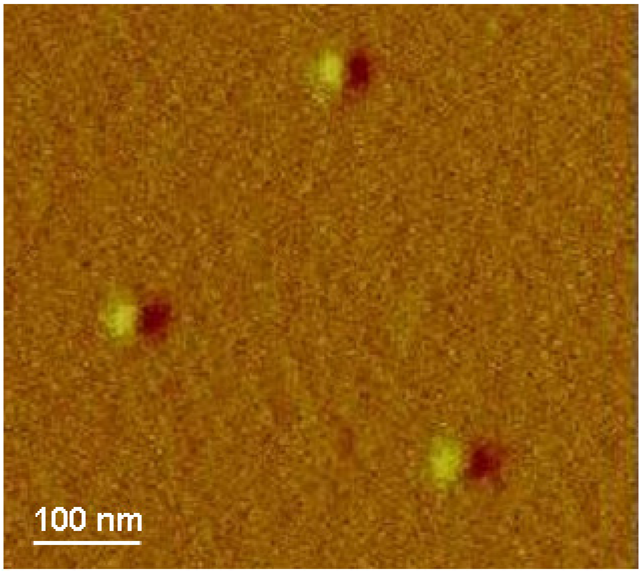

Figure S2. AFM image of LNC-NS-629. The morphology and size of the LNC-NS-629 was characterized by atomic force microscopy with a Nanoscope III Multimode scanning probe microscope (Digital Instruments).

Cathepsin B	+/+	+/-	-/-	+/+	+/-	-/-
NS-196	+	+	+	-	-	-

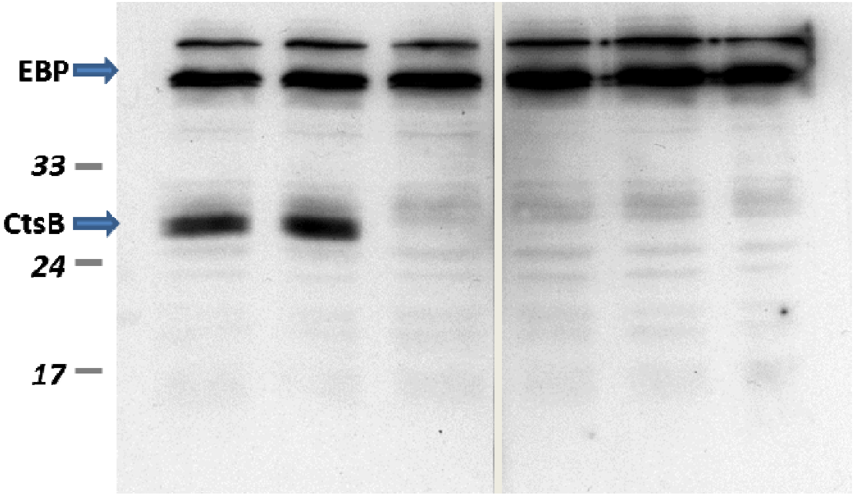

Figure S3. Detection of active membrane-bound CtsB on the surface of PyMT tumor cells. Cell surface labeling of CtsB by the biotinylated CtsB inhibitor (NS-196) was performed by incubation of PyMT tumor cells with 10 µmol/L NS-196 for 1 hour at 4º C followed by extensive washing with PBS. Western blots of cell lysates were developed with streptavidin peroxidase. CtsB expression was detected in CtsB wild-type and heterozygous CstB+/- PyMT cells, but not in CstB knock-out cells. Representative blot of three independent experiments is shown. Endogenously biotinylated proteins (EBP) served as a loading control.

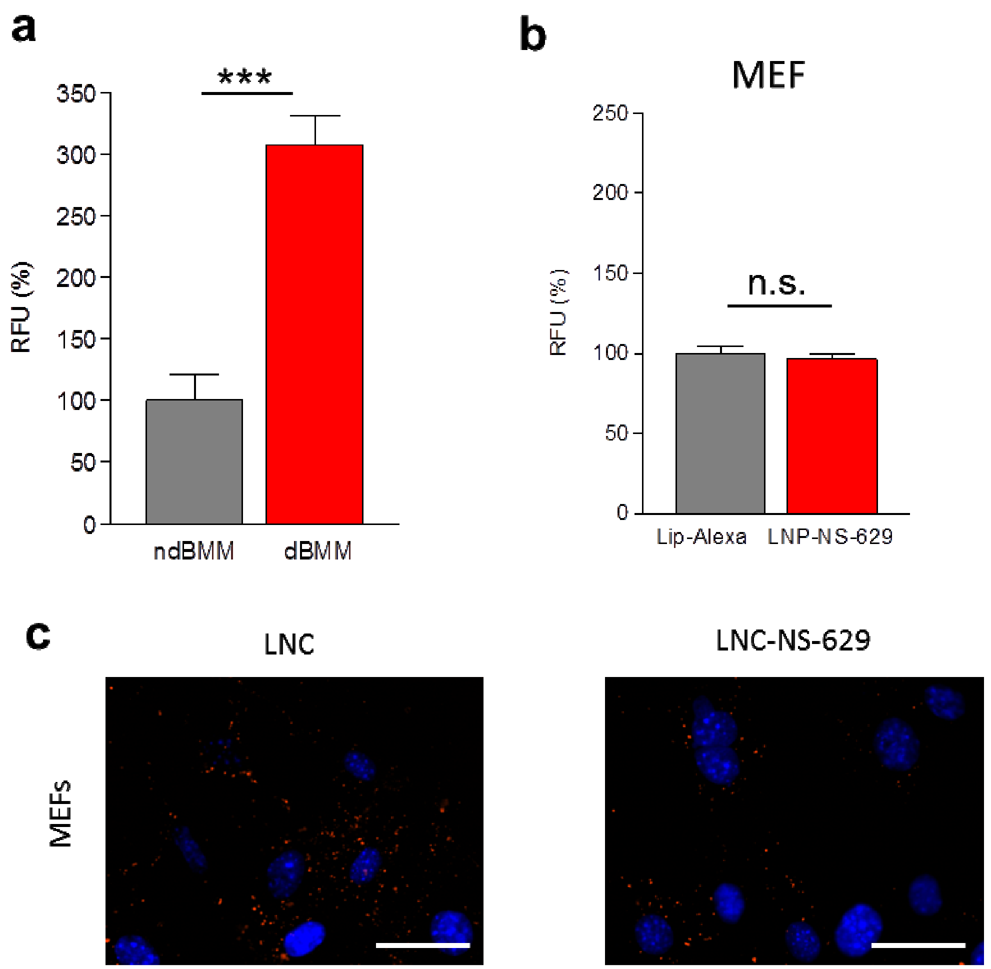

Figure S4. a) Measurement of fluorescent signal accumulated after incubation of non-differentiated bone marrow-derived macrophages (ndBMM) and differentiated bone marrow-derived macrophages (dBMM) with Alexa Fluor 555-functionalized LNC-NS-629 for 1 h at 4℃. Data are representative of three separate experiments. b) Measurement of fluorescent signal accumulated after incubation of primary mouse embryonic fibroblasts (MEFs) with Alexa Fluor 555-functionalized naked LNC and LNC-NS-629 for 1 h at 4℃. c) Fluorescence images of primary mouse embryonic macrophages (MEFs) incubated with Alexa Fluor 555-functionalized naked LNC or LNC-NS-629 for 1 h at 4 ℃. Scale bar, 20 µm.

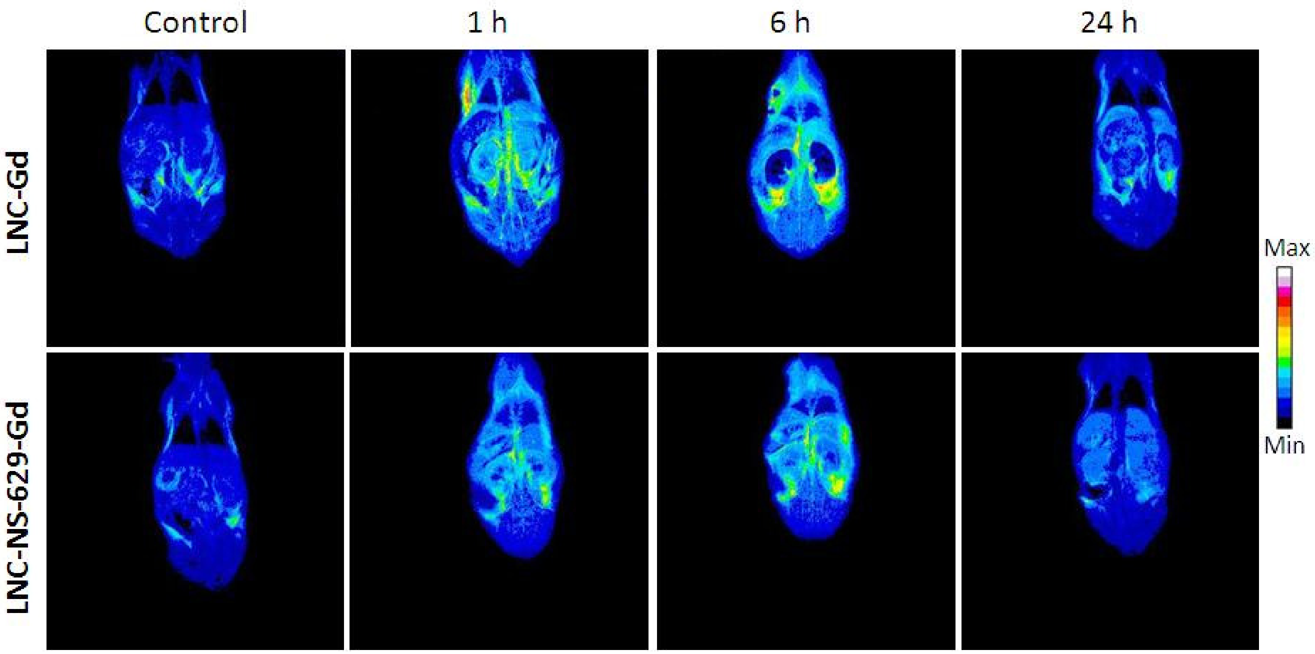

Figure S5. T_1–weighted MR images (TE=12 ms, TR=400 ms) of an FVB mouse before and 1, 6 and 24 hours after administration of LNC and LNC-NS-629 containing MRI contrast agent, Magnevist®. No accumulation of Magnevist® loaded LNC-NS-629 was detected in healthy organs.

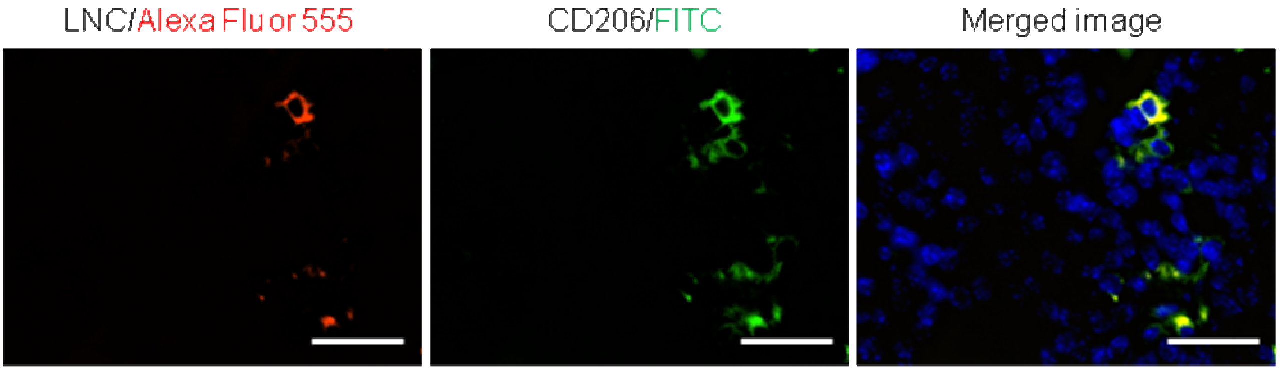

Figure S6. *In vivo* targeting of Alexa 555 (red) encapsulated into the naked liposomes (LNC). Tissues were co-stained with tumor-associated macrophages marker (CD206-FITC; green). Scale bars, 100 µm

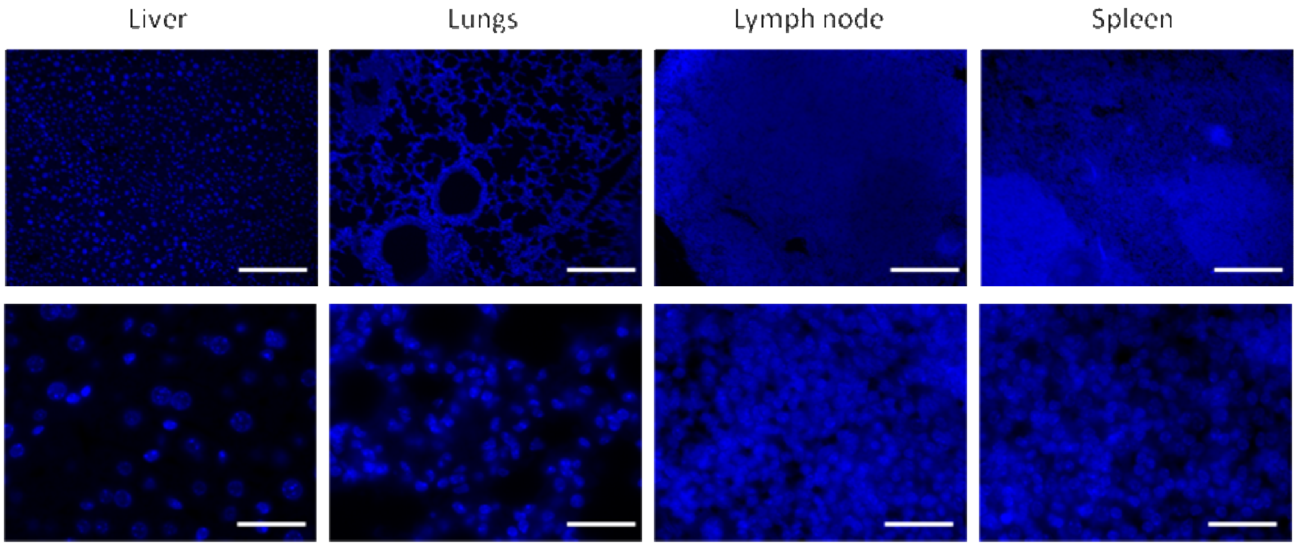

Figure S7. Fluorescence microscopy of the liver, lung, lymph node and spleen after intraperitoneally administrated Alexa Fluor 555-functionalized LNC-NS-629 liposomes. No accumulation of fluorescent cargo was detected in the analyzed tissues. The scale bar corresponds to 200 µm (top row) and 20 µm (bottom row).

Table S1. Identified peptides of cathepsin B in conditioned media from primary PyMT tumor cells (PyMT TC) and differentiated bone marrow-derived macrophages (dBMM). For all identified peptides, their corresponding peptide masses, posterior end probability scores (PEP) and peptide scores are shown in the table. Proteomic analysis was performed on conditioned media from three biological samples.

Sequence	Peptide mass [Da]	PEP	Peptide score
[1]DQGSCGSCWAFGAVEAISDR	2171.9001	2.11E-99	336.47
EIMAEIYK	995.49977	0.019179	122.13
EQWSNCPTIGQIR	1587.7464	1.17E-07	181.98
GENHCGIESEIVAGIPR	1836.8788	2.87E-23	223.76
HEAGDMMGGHAIR	1380.6027	1.70E-18	221.32
HFGYTSYSVSNSVK	1574.7365	2.12E-26	237.67
KLCGTVLGGPK	1128.6325	0.0032377	125.72
LCGTVLGGPK	1000.5376	0.00096055	134.26
NFYNVDISYLK	1374.682	1.45E-23	245.08
NGPVEGAFTVFSDFLTYK	1990.9676	1.39E-50	268.12
SCEAGYSPSYK	1247.5129	8.14E-07	177.78
SCEAGYSPSYKEDK	1619.6774	4.70E-26	241.15
TCIHTNGR	957.44505	0.014612	126.52
TDQYWGR	924.40898	0.028832	123.69
VAFGEDIDLPETFDAR	1793.8472	2.99E-13	197.19

[1] Peptide DQGSCGSCWAFGAVEAISDR was identified only in dBMM cells media.

ACKNOWLEDGEMENTS

The authors thank M. Skarabot for atomic force microscopy, A. Sepe, K. Maher, T. Maric and N. Kopitar-Jerala (Jozef Stefan Institute) for technical and methodological assistance. We are grateful to Thomas Reinheckel (Institut für Molekulare Medizin und Zellforschung, Albert-Ludvig Universitat Freiburg, Germany) for kindly provided PyMT cells. The research leading to these results was supported in part by the European Community's Seventh Framework Programme (grant agreement no. 201279 Microenvimet and grant agreement no. 280761 Alexander to O.V. and B.T., and grant agreement no. 241919 Livimode to B.T.), the Slovenian Research Agency (research grant no. P1-0140, B.T. and research grant no. J3-4267 O.V.), and the Deutsche Forschungsgemeinschaft (Heisenberg-Fellowship for N.S.).

REFERENCES

[1] N. Schaschke, I. Assfalg-Machleidt, T. Lassleben, C. P. Sommerhoff, L. Moroder, W. Machleidt, *FEBS Lett* **2000**, *482*, 91-96.

[2] O. Vasiljeva, A. Papazoglou, A. Kruger, H. Brodoefel, M. Korovin, J. Deussing, N. Augustin, B. S. Nielsen, K. Almholt, M. Bogyo, C. Peters, T. Reinheckel, *Cancer Res* **2006**, *66*, 5242-5250.

[3] J. Cox, M. Mann, *Nat Biotechnol* **2008**, *26*, 1367-1372.
